# Explainable Artificial Intelligence for Rehospitalization and Financial Burden of Fertile Women in Orthopedic Care

**DOI:** 10.3390/healthcare14010118

**Published:** 2026-01-03

**Authors:** Kwang-Sig Lee, Jaehwan Kim, Seung Beom Han

**Affiliations:** 1 AI Center, Korea University College of Medicine, Seoul 02841, Republic of Korea; ecophy@korea.ac.kr; 2 Department of Marketing, Korea University Business School, Seoul 02841, Republic of Korea; 3 Department of Orthopedic Surgery, Korea University College of Medicine, Seoul 02841, Republic of Korea

**Keywords:** artificial intelligence, rehospitalization, fertile women, medical cost

## Abstract

**Background:** Fertile women represent a socially and medically significant patient group, yet little research has examined their rehospitalization behavior and financial burden in clinical settings. This study develops predictive and explainable artificial intelligence for rehospitalization and medical costs among reproductive-age orthopedic patients. **Methods:** Electronic health records of 83 women (aged 15–49) at a major university hospital in Korea were analyzed. Six machine learning models were developed, and model performance was assessed using accuracy, the area under the curve, the root mean square error and its scaling invariant divided by the interquartile range (RMSE/IQR). Shapley Additive Explanations were applied to interpret predictors of rehospitalization. Additional analyses explored determinants of patients’ total and uncovered medical costs. **Results:** The random forest outperformed other models in predicting rehospitalization (area under the curve 0.92 vs. 0.73 for logistic regression). Key predictors included major disease, systolic blood pressure, platelet count, age, and treatment costs. The random forest also yielded lower error rates than linear regression in forecasting patients’ costs (e.g., RMSE/IQR for total cost: 1.05 vs. 1.14). Several factors—such as blood pressure, pulse, and hematocrit—were influential for both rehospitalization and costs. **Conclusions:** Predictive and explainable artificial intelligence can support medical centers in anticipating the rehospitalization and financial burden of fertile women. By integrating medical and socioeconomic determinants, hospitals may design strategies that enhance patient rehospitalization while addressing broader societal priorities in women’s health.

## 1. Introduction

For every hospital and public healthcare institution, it is a fundamental responsibility to ensure that patients—particularly those who have required inpatient care—receive appropriate treatment in a timely manner. From public health and managerial viewpoints of medical centers, fertile women represent a strategically important patient group. Their reproductive health directly affects birth rates, which in turn determine the sustainability of national demographics and economic development. For this reason, international organizations—WHO and OECD—have also emphasized that women’s health in their reproductive years should be regarded as a central priority in national health policy, guiding reforms in public healthcare and insurance systems [1,2]. They have recurring and diverse medical needs throughout pregnancy, childbirth, and child-rearing, which makes rehospitalization and long-term follow-up highly probable. Also, they often play a pivotal role in making healthcare decisions for their families, including children, spouses, and parents. Thus, managing this group not only enhances individual-level rehospitalization but also facilitates family-level acquisition, expanding the hospital’s customer base [2,3,4,5,6].

Taken together, these perspectives indicate that understanding fertile women’s interactions with hospitals and predicting their decisions to revisit or accept treatments are essential for pursuing a dual value creation strategy: achieving both primary (medical) and secondary (social) contributions. As with all patients, rehospitalization behavior in this group is influenced not only by their medical conditions but also by their demographic and socioeconomic status in complex ways.

In this context, we propose a machine learning study to develop an artificial intelligence approach that addresses the structure of this relationship and identifies the key factors that medical centers should be aware of. A recent review of 43 machine learning studies published between 2015 and 2019 shows that the random forest (16 articles, 37%), the artificial neural network (14 articles, 33%) and logistic regression (12 articles, 28%) were the most popular machine learning approaches for the prediction of hospitalization, with an average accuracy of 70%. The predictors of these 43 studies included basic information (age, gender, marriage), socioeconomic status (insurance, income, education, employment), health conditions (subjective health, major diseases, minor diseases, comorbidity, severity), vital signs, blood indicators, past history (reservation, utilization), hospitalization route (outpatient, emergency) and medical access [7]. According to another recent review of 93 machine learning studies published between 1970 and 2019, these predictors were the most representative variables in clinics data, Lisbon Portugal Data and Kalamata Greece Data [8].

However, little literature is available on predictive and explainable artificial intelligence for fertile women’s rehospitalization in medical centers. In this vein, this study develops a machine learning approach as predictive and explainable artificial intelligence to address this outcome. Specifically, we train and validate artificial intelligence models that can anticipate rehospitalization among fertile women patients as well as the financial burden (medical costs) they are expected to face.

## 2. Methods

### 2.1. Data

Data came from electronic health records for 83 orthopedics patients in a major university hospital in South Korea, who were hospitalized before 2022 and reserved for rehospitalization in 2022. These patients were fertile women between the ages of 15 and 49 [1] who needed orthopedic treatment. The key dependent variable is rehospitalization (measured via no vs. yes) in 2022. Twenty-seven predictors for the year 2022 were included for each patient: (1) record variable: rehospitalization month, reservation month; (2) basic information: age, marriage (0 unmarried, 1 married, 2 widowed, 3 divorced); (3) socioeconomic status: health insurance, employment (0 unemployed, 1 on vacation, 2/3/4 1st/2nd/3rd industry, 5 other); (4) health conditions: major disease (M code), comorbidity, severity; (5) vital signs: body temperature, pulse, respiration, systolic blood pressure, diastolic blood pressure, glucose, red blood cell, white blood cell, hemoglobin, hematocrit, platelet; (6) past history: drinking, smoking, cancelation; (7) hospitalization route: outpatient, emergency; (8) geographic factor: residential area among 10 provinces; and (9) financial burden: expected medical cost total, cost uncovered. The missing rate was lower than 10 percent and median imputation was used across board.

### 2.2. Analysis

Six machine learning models in previous healthcare studies [9,10,11,12] were used to predict rehospitalization: logistic regression, decision tree, naïve Bayes, random forest, support vector machine and artificial neural network. Their detailed explanations are given in a review article [12]. For example, a random forest is essentially an ensemble learning technique that operates by constructing a multitude of decision trees and relying on their collective decision-making to enhance predictive accuracy [12]. The 83 cases with full information were split into training and validation sets with a 75:25 ratio (62 vs. 21 cases). Criteria for the validation of a trained model were (1) accuracy for rehospitalization (the ratio of correct predictions among 21 cases), (2) the area under the curve for rehospitalization (the area under the plot of sensitivity vs. 1—specificity), (3) F1 for rehospitalization (the harmonic mean of sensitivity and specificity) and (4) the root mean square error for medical cost. For securing the validity and reliability of the outcome, the random split and the statistical analysis were repeated 50 times and their average accuracy, area under the curve and F1 were estimated for each machine learning model above. The default values of hyperparameters were used for six machine learning models. The number of trees was 1000, the criterion of split was GINI and the depth of a tree was not predetermined for the random forest. Likewise, the number of hidden layers was two, their size was 10, their activation function was the rectified linear unit, the solver of weight optimization was the limited memory Broyden–Fletcher–Goldfarb–Shanno algorithm, the L2 regularization was 0.0001 and the learning rate was 0.001.

Regarding the patient-related variables, (1) machine learning variable importance was calculated for identifying major predictors and (2) Shapley Additive Explanation (SHAP) values were derived to analyze the directions of their associations with rehospitalization. The variable importance of a predictor measures its contribution for the performance of machine learning [12]. For example, let us assume that the variable importance of a predictor employment for rehospitalization is 0.027. Here, the contribution of the predictor is 2.7% in predicting rehospitalization. The SHAP value of a predictor for a participant measures the difference between what machine learning predicts for the probability of rehospitalization with and without the predictor [12]. For instance, let us suppose that (1) the SHAP values of total cost anticipated for rehospitalization have the range of (−0.12, 0.03) and (2) their mean value is positive (e.g., 0.001). Here, some participants have SHAP values as low as −0.12, and other participants have SHAP values as high as 0.03. The inclusion of this predictor (total cost) into machine learning will decrease or increase the probability of rehospitalization by the range of −0.12 and 0.03. Then, the max value (0.03) is to be considered as a representative estimate, given that their mean value is positive (positive association). Thus, the inclusion of total cost into machine learning will increase the probability of the dependent variable (rehospitalization) by 0.03. Finally, it can be noted that R-Studio 1.3.959 (R-Studio Inc.: Boston, MA, USA) and Python 3.8.8 (Van Rossum G, Drake FL.: Scotts Valley, CA, USA) were employed for the analysis between 1 January and 31 May 2024.

## 3. Results

### 3.1. Descriptive Statistics

Table 1 presents the descriptive statistics of the variables used in model development. We briefly summarize them starting from categorical variables first. Among the 83 fertile women included in this study, 82% (68 patients) were rehospitalized, while 4% (3) had a prior cancelation history. More than half of the patients experienced rehospitalization in July or later (59%) or made their reservation from July onward (54%). In terms of socioeconomic characteristics, 57% (47 patients) were married, 90% (75) were covered by health insurance, 76% (63) were employed, and 77% (64) resided in Seoul.

Regarding health-related factors, 24% (20 patients) had a major disease, only 1% (one patient) had comorbidities, and a large majority (92%, 76 patients) were classified as having high severity. Lifestyle factors included 39% (32 patients) reporting alcohol consumption and 14% (12 patients) reporting smoking. Only one patient (1%) was admitted via emergency hospitalization.

For continuous variables, the median age was 36 years. Vital signs and laboratory measures indicated a median body temperature score of 2 (on a 0–3 scale), pulse of 41 per minute, and respiration of 7 per minute. Median blood pressure values were 122 mm Hg (systolic) and 89 mm Hg (diastolic). Median laboratory findings included glucose at 90 mg/dL, red blood cell count at 4 million/µL, white blood cell count at 6000/µL, hemoglobin at 13 g/dL, hematocrit at 39%, and platelet count at 265,000/µL. Finally, the measures for patients’ medical costs showed that the median was KRW 2640 thousand in total cost and KRW 1195 thousand in uncovered cost.

### 3.2. Predictive and Explainable Artificial Intelligence on Rehospitalization

**Figure 1:**
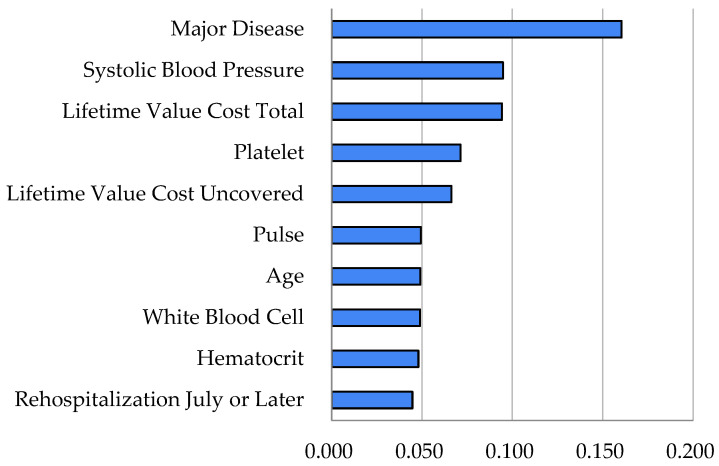
Random Forest Variable Importance Results—Rehospitalization.

Table 2 gives the outcomes of predictive artificial intelligence for the rehospitalization of fertile women patients. Here, the random forest registered a much higher area under the curve compared to logistic regression for the prediction of rehospitalization (92% vs. 73%). On the other hand, Table 3 and Figure 1 present the results of explainable artificial intelligence regarding the roles of the predictors in the random forest. Figure 1 shows their variable importance outcomes (namely, the strengths of their contributions to the performance of the random forest), whereas Table 3 shows their SHAP max (or min) values (that is, the directions of their contributions for the probability of the dependent variable (rehospitalization)). Here, rehospitalization had strong associations with the following predictors, whose variable importance outcomes were listed together with their SHAP max (or min) values for positive (or negative) associations in brackets.

We focus on the top 10 variables ranked by importance: major disease [variable importance: 0.160 (SHAP: 0.061)], systolic blood pressure [0.095 (0.028)], cost total [0.094 (0.026)], platelet [0.071 (0.023)], cost uncovered [0.066 (0.028)], pulse [0.049 (0.014)], age [0.049 (0.019)], white blood cell [0.049 (−0.128)], hematocrit [0.048 (0.012)] and rehospitalization July or later [0.045 (−0.119)]. For example, a predictor cost total made a positive contribution of 9.4% for the performance of the random forest and a positive contribution of 2.6% for the probability of the dependent variable (rehospitalization). Conversely, a predictor rehospitalization July or later made a positive contribution of 4.5% for the performance of the random forest and a negative contribution of 11.9% for the probability of the dependent variable (rehospitalization). Here, the contributions of predictors employment and residence Seoul were positive (2.7%, 1.0%) for the performance of the random forest and negative (−1.9%, −1.1%) for the probability of the dependent variable (rehospitalization).

### 3.3. Predictive and Explainable Artificial Intelligence on Medical Cost

In the next step, we turn to the patient’s financial burden in terms of medical costs, which are expenditure total and uncovered in our data. Here, the focus is not on prediction itself but on exploring which patient characteristics are associated with higher or lower costs, offering the medical center potentially useful information about how financial burden may indirectly influence its patients’ rehospitalization decisions.

**Figure 2:**
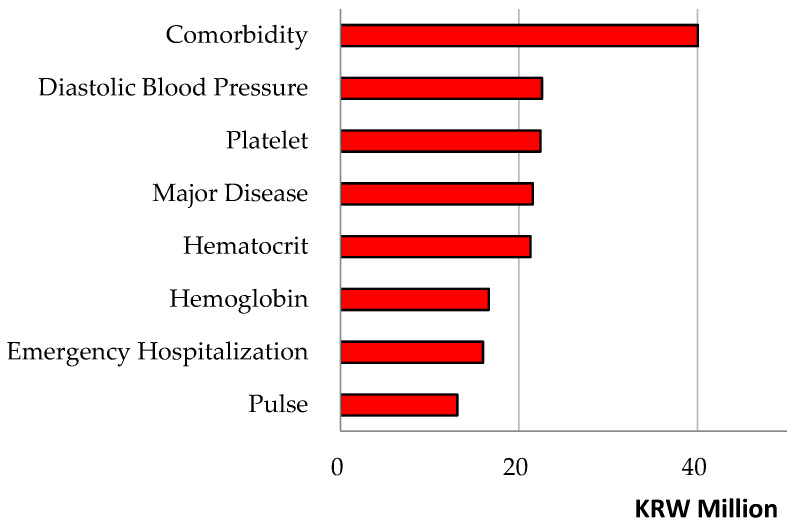
Random Forest Variable Importance Results—Cost Total.

Linear regression and random forest were employed to predict medical cost total and uncovered. The root mean square error and its scaling invariant divided by the inter-quartile range were used for measuring model performance. As the results show, the random forest showed a slightly lower root mean square error divided by the interquartile range compared to linear regression for the prediction of cost total (or uncovered), i.e., 1.05 (or 1.03) vs. 1.14 (or 1.35) in Table 4. Then, from the (random forest) variable importance outcomes and their respective (linear regression) coefficient estimate values in Table 5 and Figure 2, we can see the major features of the patients’ information about the medical cost they would bear.

**Figure 3:**
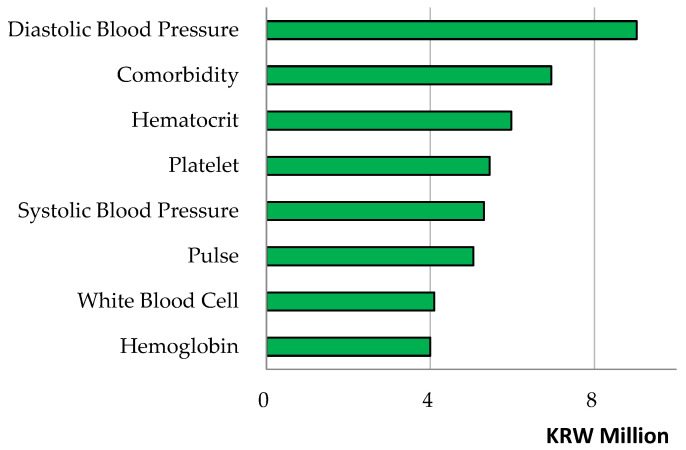
Random Forest Variable Importance Results—Cost Uncovered.

**Figure 4:**
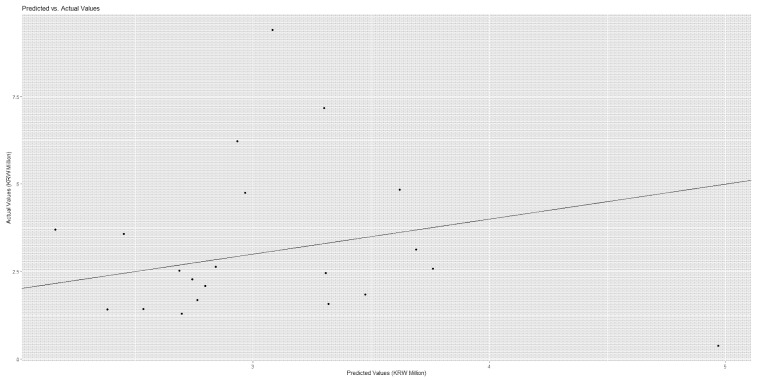
Scatter Plot—Cost Total.

**Figure 5:**
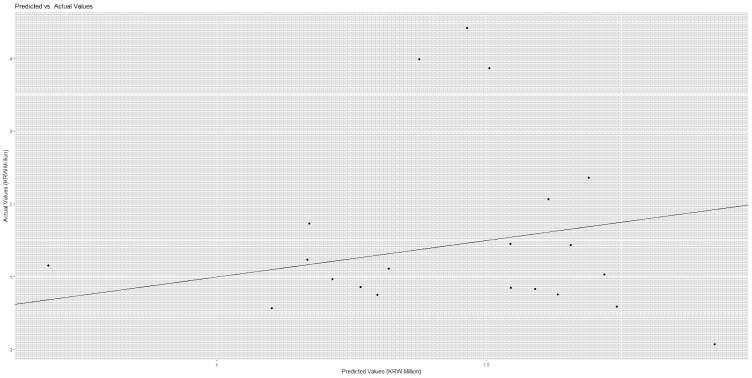
Scatter Plot—Cost Uncovered.

The medical cost ‘total’ had strong relationships with the following predictors with their respective variable importance outcomes and coefficient estimate values (divided by 1000) in brackets: comorbidity [40,047 (7838)], diastolic blood pressure [22,587 (16)], platelet [22,423 (0)], major disease [21,558 (−250)], hematocrit [21,298 (652)], hemoglobin [16,622 (−1795)], emergency hospitalization [15,974 (4421)], pulse [13,106 (43)], rehospitalization July or later [12,338 (−8)] and systolic blood pressure [11,968 (1)]. For instance, a predictor of comorbidity made a positive contribution of KRW 40,047 thousand for the performance of the random forest and it will increase total cost by KRW 7838 thousand. Conversely, a predictor rehospitalization July or later made a positive contribution of KRW 12,338 thousand for the performance of the random forest and it is likely to decrease total cost by KRW 8 thousand. Here, the contributions of predictors, employment and residence Seoul were positive (KRW 3012 thousand, KRW 2425 thousand) for the performance of the random forest and positive (KRW 20 thousand, KRW 59 thousand) for total cost. In this vein, these patients’ groups deserve due attention even though this issue is beyond the scope of this study. The results were similar in Table 6 and Figure 3 when we considered the medical cost ‘uncovered’. Figure 4 and Figure 5 show the scatter plots of actual vs. predicted costs that patients would bear. The actual and predicted costs have a perfect match on the linear line. Figure 4 and Figure 5 demonstrate that the actual and predicted costs have a good match around the linear line.

### 3.4. Explainable Artificial Intelligence Summary

In Table 7, outcomes for rehospitalization are aligned with outcomes for medical costs total and uncovered (financial burden), respectively. First, it is noteworthy that eight predictors (rehospitalization July or later, age, major disease, pulse, systolic blood pressure, white blood cell, hematocrit and platelet) ranked within the top 10 for rehospitalization, and they were informative about either cost total or cost uncovered at the same time. Indeed, these costs were top-5 predictors for rehospitalization. However, employment status and residential area (Seoul) turned out to be not important to inform medical costs. These two features were not among the top 10 predictors for rehospitalization in this study even though they are widely considered to be major predictors for rehospitalization in existing literature [7,8].

Second, it can be noted that the signs of some major predictors for rehospitalization were consistent with those of medical cost total. They are rehospitalization July or later (negative), age (positive), pulse (positive), systolic blood pressure (positive), white blood cell (negative) and hematocrit (positive). Here, the signs denote random forest SHAP means (multiplied by 100) for rehospitalization and linear regression coefficient estimates for medical cost total. These predictors can be considered to provide a managerial guideline for securing customers’ rehospitalization and anticipating their financial burden for scheduled medical services.

## 4. Discussion

### 4.1. Summary

This study developed machine learning as predictive and explainable artificial intelligence for fertile women’s rehospitalization and medical costs in a medical center. The random forest largely outperformed the other approaches. Based on random forest variable importance and Shapley Additive Explanations outcomes, patients’ rehospitalization decision can be reasonably predicted by major disease (positive), systolic blood pressure (positive), platelet (positive), pulse (positive), age (positive), employment (negative) and residence Seoul (negative). From the additional analysis to explore patients’ other characteristics that might be informative about their financial burden for rehospitalization, it is found that medical cost total had significant relationships with diastolic blood pressure (positive), comorbidity (positive), hematocrit (positive), platelet (positive), age (negative), employment (positive) and residence Seoul (positive).

### 4.2. Contributions

This study makes the following contributions to the field of medical informatics and quantitative marketing. First, the primary contribution is that this study offers artificial intelligence for predicting hospital patients’ rehospitalization based on a comprehensive set of patients’ data including both physical/medical condition and social/economic feature variables. This study also brings robust artificial intelligence for understanding patients’ financial burden to receive medical treatment via rehospitalization. As reported above, the random forest registered a much higher area under the curve compared to logistic regression for the prediction of rehospitalization (92% vs. 73%). Moreover, the random forest showed a slightly lower root mean square error divided by the interquartile range compared to linear regression for the prediction of medical cost total/uncovered (1.05/1.03 vs. 1.14/1.35). The superior performance of the random forest has been well documented. In a previous study [13] that compared the performance outcomes of 179 models based on 121 datasets from the University of California Irvine Machine Learning Repository (http://archive.ics.uci.edu/), the random forest (with max accuracy 94%) ranked first among the 179 models, followed by the support vector machine (with max accuracy 92%). This result can be attributed to the fact that the random forest is a group of decision trees making majority votes on the dependent variable (“bootstrap aggregation”) [12]. A majority vote made by 1000 doctors would be more reliable than a vote made by one doctor. Likewise, a majority vote made by 1000 decision trees would be more reliable than a vote made by a single machine learning model.

Second, this study confirms the existing literature on age and overall health as major determinants of rehospitalization among orthopedic patients. A recent study of 30-day rehospitalization among orthopedic patients reviewed 24 original studies published after 2006 [14]. Based on this review, rehospitalization had positive associations with age and American Society of Anesthesiologist score (overall health) in three or more original studies reviewed. In a similar context, another recent study of 90-day rehospitalization among orthopedic patients reviewed 16 original studies published after 2013 [15]. It was found in this review that rehospitalization had positive relationships with diabetes (Odds Ratio [*p*-value] 1.246 [0.000]) and American Society of Anesthesiologist score (1.502 [0.000]) in three or more original studies reviewed. These results are consistent with those of this study: Based on random forest variable importance outcomes in this study, rehospitalization had strong associations with age and overall health (represented by major disease, systolic blood pressure, platelet, pulse, white blood cell and hematocrit).

### 4.3. Limitations

This study comes with some limitations, which offer avenues for future research. First, this study used a small sample (83 orthopedics patients) from a single center (major university hospital). These patients were fertile women between the ages of 15 and 49 [1]. The rationale for selecting this specific group was that low birth rates are the most urgent issues in many countries including South Korea [2,3,4,5,6]. Expanding data is expected to improve the validity of this study. Second, multinomial classification was beyond the scope of this study. Multinomial classification can be defined as “combining multiple dependent variables into one and conducting its classification” in previous healthcare studies [9,16,17]. In a previous artificial intelligence study on diabetes and its comorbid conditions [9], for example, the dependent variable (disease comorbidity) was designed for four categories: “0” for diabetes no, comorbid disease no; “1” for diabetes no, comorbid disease yes; “2” for diabetes yes, comorbid disease no; and “3” for diabetes yes, comorbid disease yes. In a similar way, a medical center can be interested in managing four groups of customers by treating the probability of their rehospitalization (no vs. yes) and their expected medical cost (low vs. high), which gives rise to four management outcomes. Future research can deal with joint modeling. Third, employment status and urban residency were not top-10 predictors for rehospitalization and medical costs. This does not agree with the existing literature [7,8] and it will be a valuable contribution to resolve this inconsistency with extended data.

Fourth, reinforcement learning [18,19,20,21,22,23] was not considered in this study. Reinforcement learning is a branch of artificial intelligence which includes three elements: (1) the environment brings rewards; (2) an agent takes actions for maximum rewards; and (3) the environment changes to the next period with given probabilities [18]. The reinforcement learning agent (e.g., AlphaGo) starts like a human agent, taking actions and maximizing rewards (e.g., the chances of victory) just based on limited information in limited periods. But the reinforcement learning agent evolves far beyond the best human agent ever from the magnificent power of big data encompassing all human agents before [18]. In fact, it has been this division of reinforcement learning that has popularized the notion of artificial intelligence as intelligence similar but superior to human intelligence [18]. Reinforcement learning became popular in finance and health, given that it does not require unrealistic assumptions but delivers superior performance to traditional approaches [19,20]. These successes were replicated in business informatics such as conversational agents [21,22,23,24,25]. We expect more studies on the topics of customer rehospitalization, which is eventually to have original patients, especially fertile women segment finish their medical care as planned in other medical practice areas as well.

## 5. Conclusions

Artificial intelligence can serve as an effective decision-support system for predicting and explaining fertile women’s rehospitalization and medical costs they should bear in a medical center. This study highlights the critical importance of fertile women’s health at societal and institutional levels at the same time. At the societal level, improving their health is indispensable for fertility protection and demographic sustainability. At the institutional level, they represent a key patient segment with high potential for their own rehospitalization and broad influence over family-level healthcare decisions, underscoring the need for targeted rehospitalization strategies.

In this backdrop, our findings demonstrate that predictive and explainable artificial intelligence—particularly random forest models—offers a powerful decision support system for fertile women’s rehospitalization and financial burden. By identifying the medical and socioeconomic factors that simultaneously drive rehospitalization and cost, hospitals can design strategies that balance financial sustainability with social responsibility. Future work should extend this framework with larger and more diverse datasets, explore multi-objective learning approaches, and integrate reinforcement learning to optimize rehospitalization and medical costs in dynamic environments.

## Figures and Tables

**Table 1:** Descriptive Statistics.

Variable	Categorical		Continuous					
	%	#	Min	Q1	Median	Q3	Max	IQR
Rehospitalization	82	68						
Rehospitalization July or Later	59	49						
Reservation July or Later	54	45						
Age			15	28	36	44	49	16
Marriage	57	47						
Health Insurance	90	75						
Employment	76	63						
Major Disease	24	20						
Comorbidity	1	1						
Severity	92	76						
Body Temperature (0 Low 3 High)			1	2	2	2	3	0
Pulse (per minute)			33	33	41	49	62	16
Respiration (per minute)			3	5	7	7	7	2
Systolic Blood Pressure (mm Hg)			102	112	122	135	176	23
Diastolic Blood Pressure (mm Hg)			65	82	89	94	109	12
Glucose (mg/dL)			90	90	90	90	156	0
RBC (million/microliter)			2	4	4	4	5	0
WBC (1000/microliter)			2	5	6	8	21	3
Hemoglobin (g/dL)			9	12	13	13	15	1
Hematocrit (%)			26	37	39	40	46	3
Platelet (1000/microliter)			67	217	265	312	489	95
Drinking	39	32						
Smoking	14	12						
Cancelation History	4	3						
Emergency Hospitalization	1	1						
Residence Seoul	77	64						
MC Total (KRW 1000)			380	1893	2640	4057	13,694	2164
MC Uncovered (KRW 1000)			0	764	1195	1927	6058	1164

Note: RBC: Red Blood Cell, WBC: White Blood Cell, MC: Medical Cost, IQR: Interquartile Range.

**Table 2:** Model Performance—Rehospitalization.

	Accuracy	AUC	Specificity	Sensitivity	F1
Logistic Regression	0.73	0.73	0.26	0.93	0.60
Decision Tree	0.82	0.82	0.81	0.83	0.82
Naïve Bayes	0.68	0.68	0.23	0.87	0.55
Random Forest	0.88	0.92	0.79	0.92	0.86
SVM	0.71	0.71	0.10	0.96	0.53
ANN	0.71	0.50	0.00	1.00	0.50

Note: AUC Area Under the Curve, SVM Support Vector Machine, ANN Artificial Neural Network.

**Table 3:** Random Forest Outcomes—Rehospitalization.

Rehospitalization	VI		SHAP		
	Value	Rank	Min	Max	Sign
Major Disease	0.160	1	−0.132	0.061	0.113
Systolic Blood Pressure	0.095	2	−0.138	0.028	0.074
Medical Cost Total	0.094	3	−0.123	0.026	0.100
Platelet	0.071	4	−0.119	0.023	0.055
Medical Cost Uncovered	0.066	5	−0.075	0.028	0.004
Pulse	0.049	6	−0.177	0.014	0.039
Age	0.049	7	−0.064	0.019	0.022
White Blood Cell	0.049	8	−0.128	0.015	−0.012
Hematocrit	0.048	9	−0.106	0.012	0.036
Rehospitalization July or Later	0.045	10	−0.119	0.016	−0.024
Reservation July or Later	0.044	11	−0.061	0.011	−0.015
Diastolic Blood Pressure	0.044	12	−0.062	0.008	−0.103
Severity	0.030	13	−0.111	0.011	0.088
Employment	0.027	14	−0.019	0.013	−0.014
Hemoglobin	0.026	15	−0.039	0.014	−0.019
Red Blood Cell	0.017	16	−0.051	0.011	0.083
Respiration	0.012	17	−0.011	0.013	−0.004
Body Temperature	0.012	18	−0.138	0.004	0.122
Marriage	0.010	19	−0.008	0.006	0.016
Residence Seoul	0.010	20	−0.011	0.012	−0.048
Cancelation History	0.009	21	−0.030	0.001	0.004
Drinking	0.007	22	−0.009	0.007	−0.020
Glucose	0.006	23	−0.087	0.007	0.012
Smoking	0.006	24	−0.013	0.002	0.005
Health Insurance	0.005	25	−0.031	0.003	0.010
Emergency Hospitalization	0.004	26	−0.063	0.002	0.069
Comorbidity	0.003	27	−0.048	0.002	0.039

Note 1: VI: Variable Importance, SHAP: Shapley Additive Explanations. Note 2: Sign = Mean × 100.

**Table 4:** Model Performance—Cost Total/Uncovered.

	RMSE	RMSE/IQR
Medical Cost Total		
Linear Regression	2468	1.14
Random Forest	2283	1.05
Medical Cost Uncovered		
Linear Regression	1570	1.35
Random Forest	1200	1.03

Note: RMSE Root Mean Square Error, IQR Interquartile Range.

**Table 5:** Random Forest and Linear Regression Outcomes—Medical Cost Total.

Medical Cost Total	VI		LR	
	Value/1000	Rank	Coef/1000	*p*-Value
Comorbidity	40,047	1	7838	0.000
Diastolic Blood Pressure	22,587	2	16	0.508
Platelet	22,423	3	0	0.967
Major Disease	21,558	4	−250	0.000
Hematocrit	21,298	5	652	0.001
Hemoglobin	16,622	6	−1795	0.001
Emergency Hospitalization	15,974	7	4421	0.009
Pulse	13,106	8	43	0.030
Rehospitalization July or Later	12,338	9	−8	0.380
Systolic Blood Pressure	11,968	10	1	0.964
Reservation July or Later	11,896	11	−27	0.716
Age	11,766	12	23	0.490
Red Blood Cell	10,504	13	−1241	0.025
White Blood Cell	10,467	14	−24	0.791
Severity	9818	15	−1219	0.004
Respiration	7710	16	416	0.017
Employment	3012	17	20	0.876
Residence Seoul	2425	18	59	0.517
Marriage	1772	19	516	0.323
Smoking	1351	20	433	0.545
Body Temperature	1257	21	−401	0.465
Glucose	1061	22	3	0.626
Drinking	960	23	651	0.193
Health Insurance	885	24	−100	0.867
Cancelation History	807	25	−937	0.446

Note: VI: Random Forest Variable Importance, LR: Linear Regression.

**Table 6:** Random Forest and Linear Regression Outcomes—Medical Cost Uncovered.

Medical Cost Uncovered	VI		LR	
	Value/1000	Rank	Coef/1000	*p*-Value
Diastolic Blood Pressure	9034	1	3	0.832
Comorbidity	6951	2	2519	0.015
Hematocrit	5974	3	572	0.000
Platelet	5446	4	0	0.860
Systolic Blood Pressure	5303	5	2	0.800
Pulse	5050	6	20	0.112
White Blood Cell	4096	7	−26	0.648
Hemoglobin	3996	8	−1372	0.000
Severity	3645	9	−563	0.032
Age	3640	10	−6	0.778
Major Disease	3237	11	−112	0.008
Reservation July or Later	2622	12	42	0.381
Health Insurance	2559	13	485	0.214
Rehospitalization July or Later	2555	14	−9	0.111
Red Blood Cell	2424	15	−1051	0.004
Respiration	1952	16	286	0.012
Marriage	1927	17	796	0.022
Employment	1115	18	16	0.846
Emergency Hospitalization	696	19	607	0.561
Residence Seoul	628	20	63	0.291
Drinking	579	21	241	0.455
Glucose	494	22	1	0.893
Body Temperature	445	23	−543	0.131
Smoking	372	24	82	0.860
Cancelation History	35	25	−104	0.896

Note: VI: Random Forest Variable Importance, LR: Linear Regression.

**Table 7:** Random Forest and Linear Regression Outcomes Summary.

Variable	Rehospitalization			Cost Total			Cost Uncovered		
	Value	Rank	Sign	Value/1000	Rank	Sign	Value/1000	Rank	Sign
Rehospitalization July or Later	0.045	10	0.113	12,338	9	7838	2555	14	3
Reservation July or Later	0.044	11	0.074	11,896	11	16	2622	12	2519
Age	0.049	7	0.100	11,766	12	0	3640	10	572
Marriage	0.010	19	0.055	1772	19	−250	1927	17	0
Health Insurance	0.005	25	0.004	885	24	652	2559	13	2
Employment	0.027	14	0.039	3012	17	−1795	1115	18	20
Major Disease	0.160	1	0.022	21,558	4	4421	3237	11	−26
Comorbidity	0.003	27	−0.012	40,047	1	43	6951	2	−1372
Severity	0.030	13	0.036	9818	15	−8	3645	9	−563
Body Temperature	0.012	18	−0.024	1257	21	1	445	23	−6
Pulse	0.049	6	−0.015	13,106	8	−27	5050	6	−112
Respiration	0.012	17	−0.103	7710	16	23	1952	16	42
Systolic Blood Pressure	0.095	2	0.088	11,968	10	−1241	5303	5	485
Diastolic Blood Pressure	0.044	12	−0.014	22,587	2	−24	9034	1	−9
Glucose	0.006	23	−0.019	1061	22	−1219	494	22	−1051
Red Blood Cell	0.017	16	0.083	10,504	13	416	2424	15	286
White Blood Cell	0.049	8	−0.004	10,467	14	20	4096	7	796
Hemoglobin	0.026	15	0.122	16,622	6	59	3996	8	16
Hematocrit	0.048	9	0.016	21,298	5	516	5974	3	607
Platelet	0.071	4	−0.048	22,423	3	433	5446	4	63
Drinking	0.007	22	0.004	960	23	−401	579	21	241
Smoking	0.006	24	−0.020	1351	20	3	372	24	1
Cancelation History	0.009	21	0.012	807	25	651	35	25	−543
Emergency Hospitalization	0.004	26	0.005	15,974	7	−100	696	19	82
Residence Seoul	0.010	20	0.010	2425	18	−937	628	20	−104
Medical Cost Total	0.094	3	0.069						
Medical Cost Uncovered	0.066	5	0.039						

## Data Availability

The original contributions presented in this study are included in the article. Further inquiries can be directed to the corresponding authors.
